# Does timing matter? Early postoperative Gamma Knife radiosurgery within 14 days is associated with longer overall survival after brain metastasis resection

**DOI:** 10.1007/s11060-026-05866-z

**Published:** 2026-11-11

**Authors:** Sarthak Sinha, Victor Goulenko, Neil D. Almeida, Venkatesh Shankar Madhugiri, Babar Gulzar, Danil Ratnikov, Aarav Trivedi, Lindsay Lipinski, Andrew Fabiano, Kenneth Snyder, Robert Fenstermaker, Robert J. Plunkett, Dheerendra Prasad

**Affiliations:** 1 Department of Radiation Medicine, Roswell Park Comprehensive Cancer Center, Buffalo, NY USA; 2 Department of Radiation Oncology, Jacobs School of Medicine and Biomedical Sciences, University at Buffalo, Buffalo, NY USA; 3 Gilman School, Baltimore, MD USA; 4 Department of Neurosurgery, Roswell Park Comprehensive Cancer Center, Buffalo, NY USA; 5 Department of Neurosurgery, Jacobs School of Medicine and Biomedical Sciences, University at Buffalo, Buffalo, NY USA; 6 Jacobs School of Medicine and Biomedical Sciences, University at Buffalo, Buffalo, NY USA; 7 Roswell Park Comprehensive Cancer Center, Elm and Carlton Streets, Buffalo, NY USA

**Keywords:** Brain metastases, Gamma Knife radiosurgery, Postoperative radiosurgery, Stereotactic radiosurgery, Resection cavity, Treatment timing, Overall survival

## Abstract

**Background:**

The optimal timing of postoperative Gamma Knife radiosurgery (GKRS) following brain metastasis resection remains uncertain. We evaluated the association between surgery-to-GKRS timing and overall survival (OS) and local recurrence-free survival (LRFS).

**Methods:**

We retrospectively analyzed 182 patients who underwent initial postoperative cavity-directed GKRS following brain metastasis resection between May 2007 through February 2025. Surgery-to-GKRS timing was evaluated continuously and using outcome-oriented cutpoint analysis across intervals of 7–60 days. Survival outcomes were assessed using Kaplan–Meier analysis and multivariable Cox regression, with additional sensitivity analyses evaluating cutpoint stability and potential confounding.

**Results:**

The median surgery-to-GKRS interval was 19.5 days. Treatment within 14 days demonstrated the greatest observed OS separation, with median survival of 37.8 versus 15.3 months for patients treated later (log-rank *p* = 0.0123). This association persisted after multivariable adjustment (HR 0.62, 95% CI 0.38–1.00; *p* = 0.0491) and additional adjustment for calendar year of surgery (HR 0.60, 95% CI 0.37–0.97; *p* = 0.037). However, the 14-day threshold did not retain statistical significance after adjustment for outcome-oriented cutpoint selection (*p* = 0.181). GKRS timing was not significantly associated with LRFS.

**Conclusions:**

GKRS within 14 days of brain metastasis resection was associated with substantially longer overall survival, highlighting the potential clinical importance of timely postoperative radiosurgical care. Although the optimal treatment interval remains uncertain, these findings support early referral, efficient multidisciplinary coordination, and minimizing avoidable treatment delays.

**Supplementary Information:**

The online version contains supplementary material available at https://doi.org/10.1007/s11060-026-05866-z.

## Introduction

Brain metastases are a common and clinically consequential manifestation of systemic cancer [1]. Surgical resection remains important for large, symptomatic, or mass-effect-producing lesions, particularly when tissue diagnosis or rapid neurologic decompression is required [2]. However, surgery alone carries a substantial risk of recurrence within the postoperative cavity, supporting the use of adjuvant radiation in appropriately selected patients [3].

Postoperative stereotactic radiosurgery (SRS) to the resection cavity has become a widely used strategy for improving local control while avoiding the neurocognitive toxicity associated with whole-brain radiotherapy [4–6]. Despite broad acceptance of postoperative cavity SRS, the optimal timing of treatment after surgery remains uncertain. Earlier treatment may reduce the interval during which residual microscopic disease remains untreated, whereas a short delay may allow postoperative recovery and evolution of the resection cavity before target delineation and treatment [7].

Prior studies have reported conflicting results regarding the optimal treatment interval. Iorio-Morin et al. found increased local recurrence with longer surgery-to-SRS delay and recommended Gamma Knife radiosurgery (GKRS) within approximately 3 weeks of resection [8]. In contrast, Yaghi et al. reported improved outcomes when postoperative radiotherapy was initiated after 22 days, while delays beyond 40 days were associated with greater progression risk [9]. More recently, Bander et al. identified an interval of approximately 22–30 days as potentially balancing local failure and posttreatment radiation effects [10]. These divergent findings leave the clinically relevant treatment window uncertain, particularly for Gamma Knife-based postoperative radiosurgery.

We therefore performed a retrospective single-institution analysis of patients undergoing postoperative GKRS after resection of brain metastases. We sought to identify the surgery to GKRS interval most strongly associated with overall survival and to determine whether this threshold was also associated with local recurrence-free survival. We hypothesized that earlier postoperative GKRS would be associated with improved outcomes and could help define a practical treatment window for radiosurgical referral and planning.

## Materials and methods

### Study design and patient selection

We conducted a retrospective, single-institution cohort study of patients with surgically resected brain metastases who underwent initial postoperative cavity-directed GKRS at Roswell Park Comprehensive Cancer Center. The study was approved by the Institutional Review Board of Roswell Park Comprehensive Cancer Center (BDR 280026), with a waiver of informed consent.

Patients were identified through a retrospective review of institutional clinical and radiosurgical records covering May 2007 through February 2025.Eligible patients had pathologically confirmed brain metastases and underwent surgical resection followed by their first GKRS treatment directed to the postoperative resection cavity. Patients were included irrespective of the surgery-to-GKRS interval, provided treatment dates and survival information were available. The primary unit of analysis was the patient. For patients with multiple resection cavities or GKRS targets, each patient was included once in survival analyses to avoid treating correlated lesions as independent observations. When multiple cavities were present, the largest postoperative cavity was used for cavity-specific treatment variables, and the total number of cavities or GKRS targets was recorded when available.

### Clinical and treatment data collection

Demographic, clinical, oncologic, and treatment-related variables were extracted from the electronic medical record and institutional Gamma Knife treatment records. Baseline variables included age at GKRS, sex, primary tumor histology, surgery date, GKRS date, preoperative functional status, functional status at GKRS, date of last clinical follow-up, and date of death when applicable. Functional status was recorded using Karnofsky Performance Status (KPS) and Eastern Cooperative Oncology Group (ECOG) performance status when available. Extracranial disease was defined as the presence of systemic metastatic disease from the primary malignancy outside the central nervous system at the time of GKRS.

Treatment variables included surgery-to-GKRS interval, GKRS prescription dose, treatment volume, prescription isodose, minimum dose, maximum dose, mean dose, beam-on time, and use of single-fraction versus hypofractionated treatment. The number of treated cavities or GKRS targets was recorded when available.

### Gamma Knife radiosurgery technique

GKRS was delivered using Gamma Knife platforms available at the institution during the study period, with frame-based or mask-based immobilization according to clinical appropriateness. The postoperative resection cavity was contoured as the primary target. Adjacent involved dura or residual enhancing disease was included when clinically indicated. Additional intact brain metastases could be treated during the same GKRS session at the discretion of the treating team.

Prescription dose and fractionation were individualized based on cavity size, target volume, lesion location, proximity to critical structures, postoperative anatomy, and team judgment. Patients were treated with either single-fraction GKRS or hypofractionated stereotactic radiosurgery.

Single-fraction GKRS was delivered in 157 patients, with prescription doses ranging from 10 to 20 Gy. Hypofractionated GKRS was delivered in 23 patients, with recorded prescription doses ranging from 21 to 35 Gy. Among the 21 patients with documented fraction counts, 20 received three fractions and one received seven fractions. The number of fractions was not documented for two additional patients who received hypofractionated GKRS. Across the 180 patients with available dosimetric data, treated volumes ranged from 0.126 to 57.327 cc, with a median of 8.25 cc. Individual fraction delivery dates and intervals between fractions were not systematically recorded in the analytical dataset.

### Outcome definitions

The primary outcome was overall survival (OS), defined as the time from GKRS to death from any cause. Patients alive at last follow-up were censored at the date of last known clinical follow-up.

The secondary outcome was local recurrence-free survival (LRFS), defined as the time from GKRS to radiographic recurrence involving the treated postoperative cavity. Patients without documented local recurrence were censored at the date of last available brain MRI. Patients were considered evaluable for LRFS if they had documented local recurrence or at least one recorded post-GKRS follow-up brain MRI. Patients without documented local recurrence or evaluable post-GKRS intracranial imaging follow-up were excluded from the LRFS analysis.

Local recurrence was identified based on follow-up contrast-enhanced brain MRI findings, incorporating the interpreting radiologist’s report and review of the imaging by the treating neurosurgeon and radiation oncologist. Suspected recurrence was evaluated in the context of available serial imaging and clinical findings to distinguish tumor progression from treatment-related changes. In more recent years, treatment response assessment mapping (TRAM) imaging was also used in selected cases to assist in differentiating radiation necrosis from tumor progression. However, advanced imaging techniques were not uniformly available or systematically applied throughout the study period.

Radiation necrosis was identified from available radiographic and clinical documentation. Treatment-related changes were characterized descriptively according to documented clinical severity and management, including observation or corticosteroid escalation. CTCAE grades were reported only when supported by the available records.

### Statistical analysis

Descriptive statistics were used to summarize baseline and treatment characteristics. Continuous variables were reported as medians with interquartile ranges, and categorical variables as frequencies and percentages. Comparisons between timing groups were performed using the Wilcoxon rank-sum test for continuous variables and the chi-square or Fisher exact test for categorical variables, as appropriate.

The surgery-to-GKRS interval was evaluated as a continuous variable and through cohort-specific cutpoint analysis using an outcome-oriented log-rank approach [11]. Candidate cutpoints from 7 to 60 days after surgery were assessed for OS, and the threshold associated with the strongest survival separation was selected for the primary dichotomized timing analysis.

To assess the robustness of the outcome-derived timing threshold, additional exploratory analyses were performed. A permutation-based maximally selected log-rank analysis using 20,000 permutations was conducted to account for searching across candidate thresholds from 7 to 60 days. Threshold stability was assessed using 5,000 patient-level bootstrap resamples, with a minimum group size of 20% required for each comparison. Additionally, restricted cubic spline Cox regression was performed to investigate the continuous association between GKRS timing and OS, adjusting for the clinical and treatment-related covariates included in the primary multivariable model. Overall timing associations and nonlinear components were evaluated using likelihood-ratio tests.

OS and LRFS were estimated using the Kaplan–Meier method and compared using the log-rank test. Cox proportional hazards regression was used to evaluate associations between GKRS timing and survival outcomes. Multivariable models sequentially adjusted for clinically relevant available covariates, including age at GKRS, sex, KPS at GKRS, prescription dose, treatment volume, single-fraction versus hypofractionated treatment, and extracranial disease status. An expanded model additionally adjusted for primary tumor histology. Calendar year of surgery was additionally included as a continuous covariate in a sensitivity analysis to assess potential confounding by temporal changes in clinical practice. Patients with missing covariate data were excluded from complete-case multivariable models. The proportional hazards assumption was evaluated using scaled Schoenfeld residual-based diagnostics, with rank-transformed event time used to assess individual covariates and the overall model.

A sensitivity analysis excluding patients with surgery-to-GKRS intervals greater than 60 days was performed to evaluate the influence of extreme delays. As an additional sensitivity analysis, OS was recalculated from the date of surgical resection rather than from the date of GKRS to determine whether the timing association was robust to the survival time origin. All statistical tests were two-sided, and p values < 0.05 were considered statistically significant.

## Results

### Cohort characteristics

A total of 182 patients with surgically resected brain metastases treated with postoperative GKRS were included (Table 1). The median age at GKRS was 63 years (IQR, 56–70), and 94 patients (51.6%) were female. The most common primary histology was non-small cell lung cancer (*n* = 80, 44.0%), followed by melanoma (*n* = 26, 14.3%), breast cancer (*n* = 22, 12.1%), renal cell carcinoma (*n* = 13, 7.1%), small cell lung cancer (*n* = 10, 5.5%), and other histologies (*n* = 31, 17.0%).

**Table 1:** Overall cohort characteristics

Characteristic	Overall cohort, *n* = 182
Age at GKRS, median (IQR), years	63 (56–70)
Female sex, n (%)	94 (51.6)
Male sex, n (%)	88 (48.4)
Primary histology, n (%)	
Non-small cell lung cancer	80 (44.0)
Melanoma	26 (14.3)
Breast cancer	22 (12.1)
Renal cell carcinoma	13 (7.1)
Small cell lung cancer	10 (5.5)
Other histologies	31 (17.0)
Surgery-to-GKRS interval, median (IQR), days	19.5 (14–29)
Surgery-to-GKRS interval, range, days	5–460
Extracranial disease present at GKRS, n (%)	39 (21.4)
Clinical follow-up from GKRS, median (IQR), months	14.4 (7.4–38.2)
Clinical follow-up from GKRS, range, months	0–170.3
Follow-up among surviving patients, median, months	30.1
KPS at GKRS, median (IQR)	80 (70–90), *n* = 142
ECOG at GKRS, median (IQR)	1 (0–1), *n* = 143
GKRS prescription dose, median (IQR), Gy	18 (18–18), *n* = 180
Treated GKRS volume, median (IQR), cc	8.25 (5.11–16.07), *n* = 180
Single-fraction GKRS, n (%)	157 (86.3)
Hypofractionated GKRS, n (%)	23 (12.6)
Fractionation unavailable, n (%)	2 (1.1)
Deaths during follow-up, n (%)	132 (72.5)
Local recurrence, n (%)	50 (27.5)

Values are reported as median (IQR) or n (%). Abbreviations: GKRS = Gamma Knife radiosurgery; KPS = Karnofsky Performance Status; ECOG = Eastern Cooperative Oncology Group

The median interval from surgical resection to GKRS was 19.5 days (IQR, 14–29; range, 5–460). Extracranial disease was present at the time of GKRS in 39 patients (21.4%). Median observed clinical follow-up from GKRS to death or last known follow-up was 14.4 months (IQR, 7.4–38.2; range, 0–170.3), and median follow-up among surviving patients was 30.1 months. Two patients had no documented clinical follow-up beyond the date of GKRS and were censored at time zero, accounting for the minimum recorded follow-up duration of zero months. Functional status at GKRS was available for most patients, with median KPS of 80 (IQR, 70–90; *n* = 142) and median ECOG performance status of 1 (IQR, 0–1; *n* = 143). Median GKRS prescription dose was 18 Gy (IQR, 18–18; *n* = 180), and median treated volume was 8.25 cc (IQR, 5.11–16.07; *n* = 180). Single-fraction GKRS was used in 157 patients, hypofractionated treatment was used in 23 patients, and fractionation information was unavailable for 2 patients.

During follow-up, 132 patients died and 50 patients developed documented local recurrence involving the treated postoperative cavity.

### Surgery-to-GKRS timing distribution and cutpoint identification

**Fig. 1:**
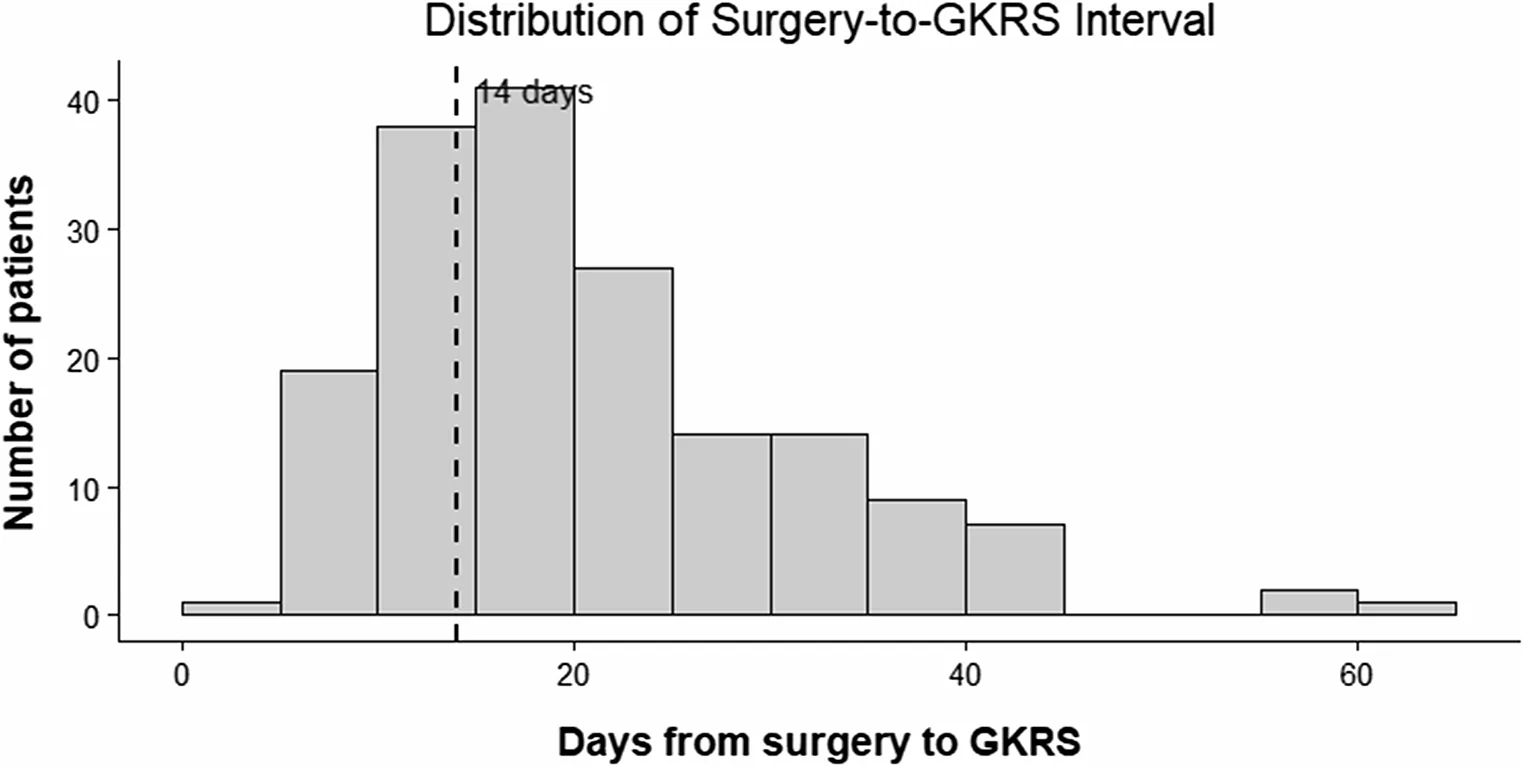
Distribution of postoperative timing from surgery to GKRS. Histogram showing the surgery-to-GKRS interval, displayed through 65 days for visualization of the predominant postoperative treatment window. The dashed line marks the 14-day threshold identified in subsequent cutpoint analysis

The median interval from surgery to GKRS was 19.5 days (IQR, 14–29 days; range, 5–460 days), with most patients treated within the first postoperative month (Fig. 1).

**Fig. 2:**
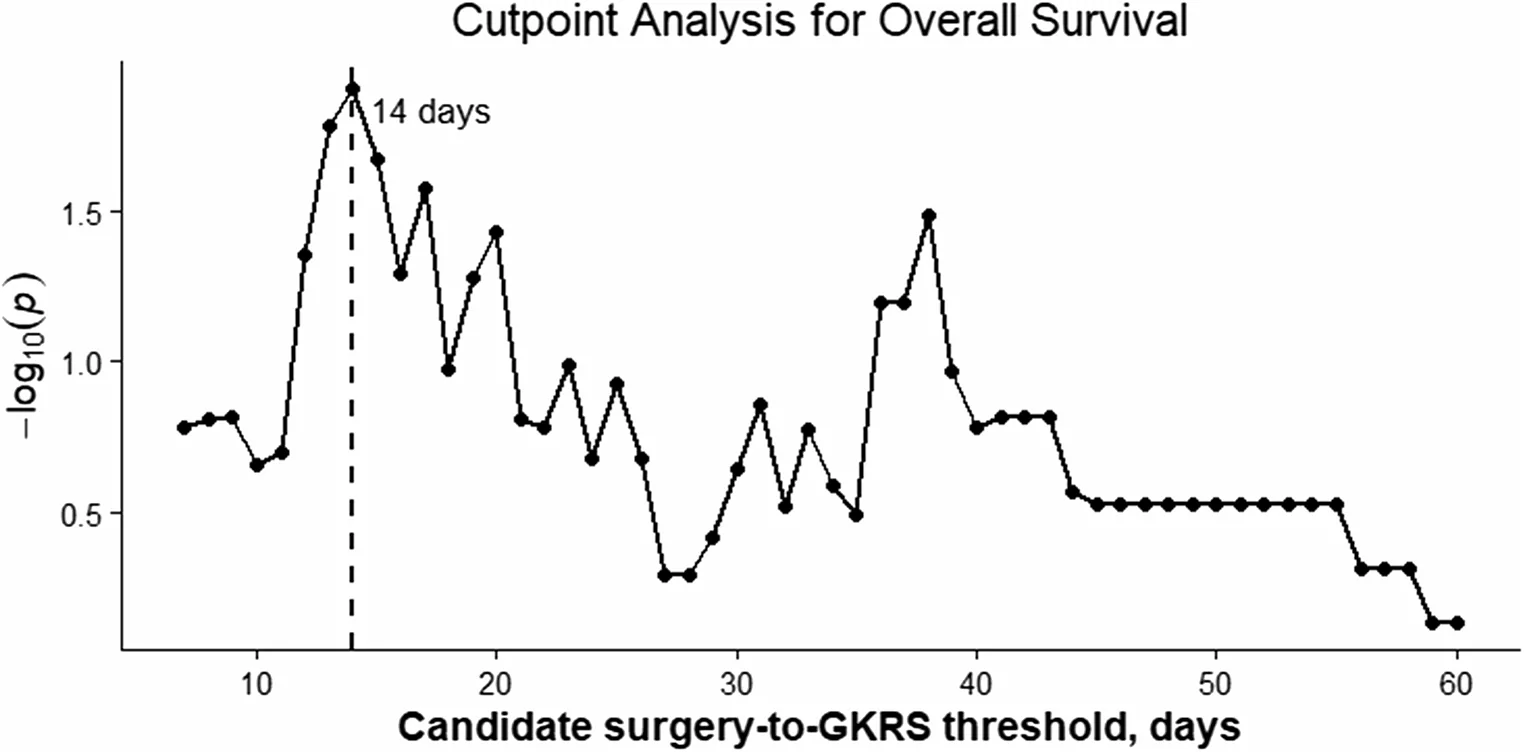
Cutpoint analysis identifying the postoperative timing threshold most strongly associated with OS. Candidate thresholds from 7 to 60 days were evaluated using log-rank testing. The 14-day threshold demonstrated the strongest separation in OS

Candidate cutpoints from 7 to 60 days after surgery were evaluated using log-rank testing for OS. The 14-day threshold, defined as GKRS delivered ≤ 14 days versus > 14 days after surgery, demonstrated the strongest OS separation among tested cutpoints (*p* = 0.0123; Fig. 2). Adjacent thresholds also showed significant separation, including ≤ 13 days (*p* = 0.0164), ≤ 15 days (*p* = 0.0211), and ≤ 17 days (*p* = 0.0265), supporting an early postoperative window centered around two weeks.

**Fig. 3:**
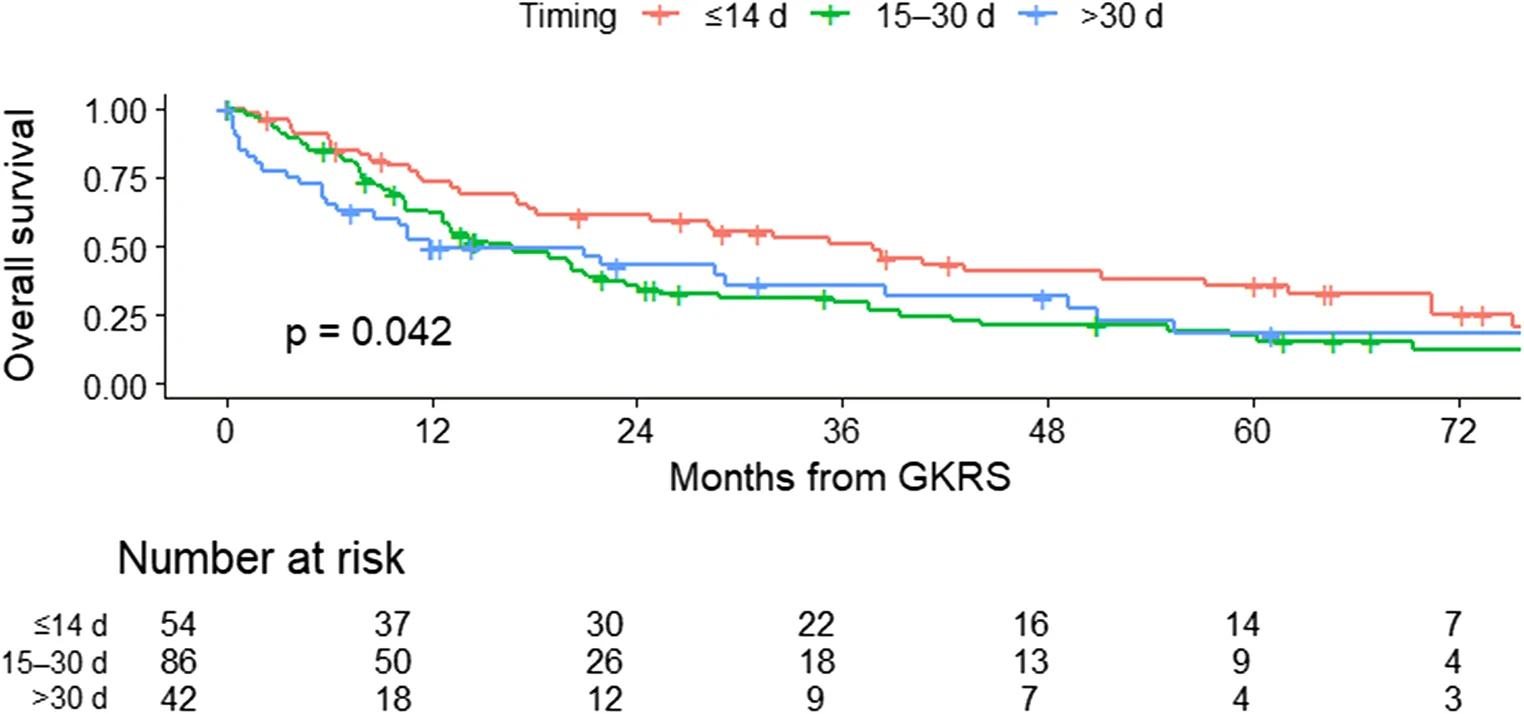
Overall survival across clinically relevant postoperative GKRS timing intervals. Kaplan–Meier curve comparing GKRS delivered ≤ 14 days, 15–30 days, and > 30 days after surgery. Overall survival differed significantly across timing groups (global log-rank *p* = 0.0418), with the longest median OS observed in patients treated ≤ 14 days

Clinically grouped timing analyses further supported this threshold. In a three-group analysis, median OS was 37.8 months for ≤ 14 days, 16.7 months for 15–30 days, and 12.0 months for > 30 days (global log-rank *p* = 0.0418; Fig. 3). Pairwise comparisons favored ≤ 14 days over both 15–30 days (*p* = 0.0179) and > 30 days (*p* = 0.0365), while OS did not differ between 15 and 30 and > 30 days (*p* = 0.801). In univariable Cox regression, GKRS at 15–30 days (HR, 1.60; 95% CI, 1.06–2.41; *p* = 0.0242) and > 30 days (HR, 1.70; 95% CI, 1.04–2.76; *p* = 0.0332) were each associated with higher mortality risk compared with ≤ 14 days.

A four-group sensitivity analysis separating 15–21 days and 22–30 days did not identify a survival advantage for the 22–30-day interval, although the global comparison across all four groups did not reach statistical significance (*p* = 0.0939; Supplementary Figure 1). Based on the cutpoint and complementary grouped analyses, the 14-day threshold was selected for the primary dichotomized timing analysis.

Additional analyses were performed to assess the robustness of the identified timing threshold. Although 14 days demonstrated the strongest observed OS separation, the association did not retain statistical significance after permutation adjustment for the outcome-oriented cutpoint search (adjusted *p* = 0.181). Nevertheless, bootstrap resampling demonstrated a concentration of selected thresholds within the early postoperative period. The 14-day threshold was the most frequently selected individual cutoff (27.3%), with 57.5% of bootstrap samples identifying thresholds between 12 and 16 days and 84.9% identifying thresholds between 10 and 21 days. These findings suggest that the observed survival separation was frequently concentrated around the first two postoperative weeks, although the precise optimal threshold remains uncertain. Complementary restricted cubic spline analysis demonstrated an overall association between timing and OS in the fully adjusted cohort (*p* = 0.0459), without statistically significant evidence of nonlinearity (*p* = 0.1636). The overall association was not statistically significant after excluding intervals greater than 60 days (*p* = 0.2408), and no discrete inflection point at 14 days was established (Supplementary Figure 4).

### Baseline and treatment characteristics by timing group

Baseline and treatment characteristics were compared between patients treated within 14 days and those treated more than 14 days after surgery (Table 2). Fifty-four patients (29.7%) underwent GKRS within 14 days, and 128 patients (70.3%) underwent GKRS after 14 days.

**Table 2:** Baseline and treatment characteristics by 14-day timing group

Characteristic	≤ 14 days, *n* = 54	> 14 days, *n* = 128	*p* value
Age at GKRS, median, years	59.5	65	0.012
Sex, n (%)			0.008
Female	36 (66.7)	58 (45.3)	
Male	18 (33.3)	70 (54.7)	
KPS at GKRS, median	80	80	0.816
Preoperative KPS, median (IQR)	80 (70–90), *n* = 38	80 (70–90), *n* = 87	0.203
ECOG at GKRS, median	1	1	0.887
Primary histology distribution	—	—	0.916
Extracranial disease present, n (%)	8 (14.8)	31 (24.2)	0.158
GKRS prescription dose, median, Gy	18	18	0.015
Treated GKRS volume, median, cc	11.92	7.79	0.041
Hypofractionated GKRS, n (%)	12 (22.2)	11 (8.6)	0.013

Continuous variables were compared using the Wilcoxon rank-sum test. Categorical variables were compared using chi-square or Fisher exact testing, as appropriate

Patients treated within 14 days were younger (median age, 59.5 vs. 65 years; Wilcoxon rank-sum *p* = 0.012) and more frequently female (66.7% vs. 45.3%; chi-square *p* = 0.008). Functional status was similar between groups, with no significant difference in median KPS (80 vs. 80; *p* = 0.816) or ECOG performance status (1 vs. 1; *p* = 0.887). Preoperative KPS was available for 125 patients and was comparable between the ≤ 14-day and > 14-day groups (median 80 [IQR, 70–90] in both groups; *p* = 0.203). Primary tumor histology distribution did not differ significantly between timing groups (*p* = 0.916). Extracranial disease was present in 14.8% of patients treated within 14 days and 24.2% of those treated after 14 days, without a statistically significant difference between groups (*p* = 0.158).

Median prescription dose was 18 Gy in both groups, although the dose distribution differed by timing group (*p* = 0.015). Median treated volume was larger in the ≤ 14-day group (11.92 vs. 7.79 cc; *p* = 0.041). Hypofractionated GKRS was used in 22.2% of patients treated within 14 days and 8.6% of those treated after 14 days (*p* = 0.013).

### Overall survival by timing group

In the overall cohort, median OS from GKRS was 19.9 months. Estimated OS rates were 82.1% at 6 months, 62.4% at 1 year, 44.8% at 2 years, 37.0% at 3 years, and 23.4% at 5 years.

**Fig. 4:**
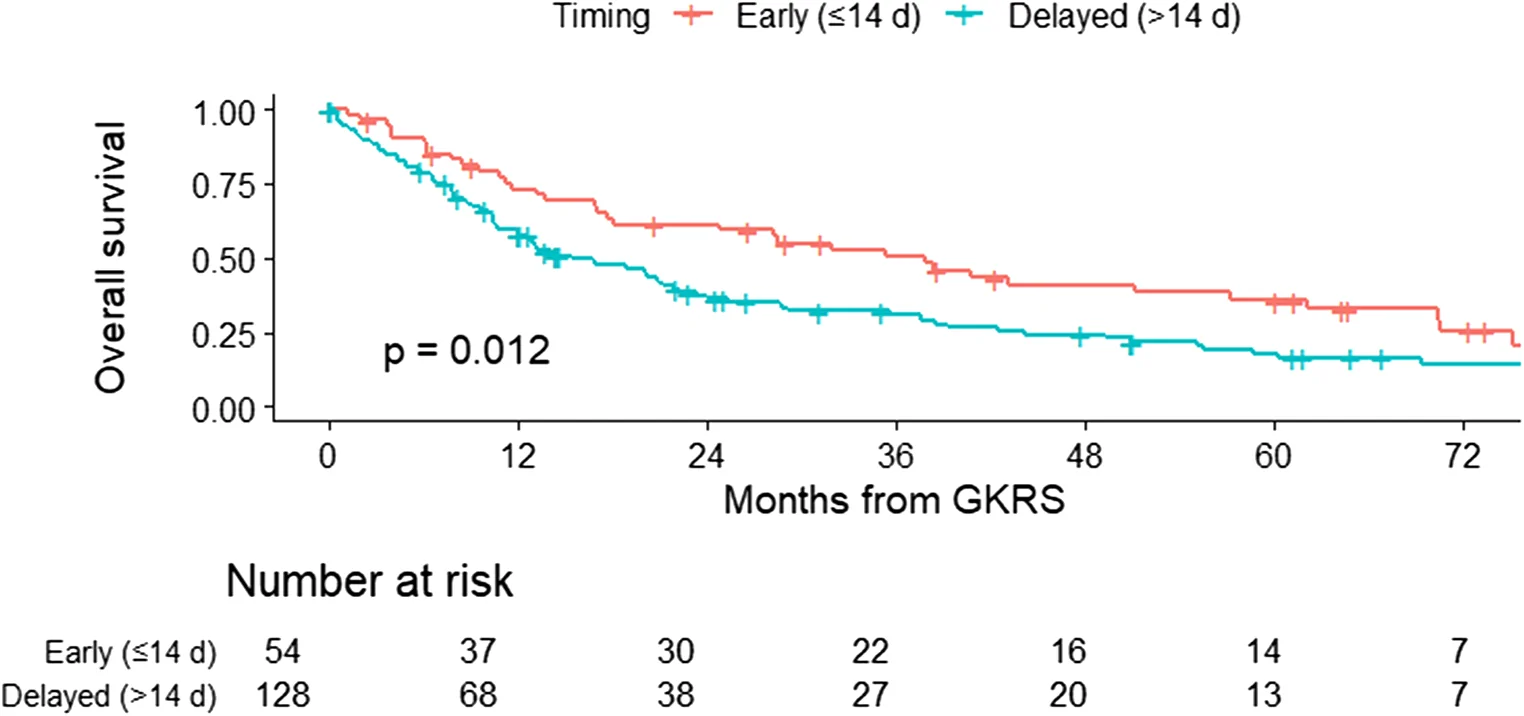
Overall survival stratified by postoperative GKRS timing. Kaplan–Meier curve comparing patients treated ≤ 14 days versus > 14 days after surgery. Patients treated ≤ 14 days demonstrated significantly longer OS (log-rank *p* = 0.0123)

Using the 14-day threshold identified in the cutpoint analysis, patients treated within 14 days demonstrated significantly longer OS than those treated more than 14 days after surgery (Fig. 4). Median OS was 37.8 months in the ≤ 14-day group compared with 15.3 months in the > 14-day group. Estimated 1-year OS was 73.2% versus 57.8%, 2-year OS was 61.3% versus 37.5%, and 5-year OS was 35.8% versus 17.7%, respectively. The difference in OS was statistically significant by log-rank testing (*p* = 0.0123).

In univariable Cox regression, GKRS within 14 days was associated with reduced mortality risk compared with GKRS after 14 days (HR, 0.61; 95% CI, 0.42–0.90; *p* = 0.0131).

### Multivariable cox regression for overall survival

The association between early postoperative GKRS and OS was evaluated using sequential multivariable Cox regression models (Table 3). After adjustment for age and sex, the association was attenuated and did not reach statistical significance (HR, 0.69; 95% CI, 0.46–1.02; *p* = 0.0613). After additional adjustment for KPS at GKRS, treatment within 14 days was associated with lower mortality risk (HR, 0.61; 95% CI, 0.38–0.98; *p* = 0.0415).

**Table 3:** Cox regression models for overall survival

Model	Covariates	*n*	HR for GKRS ≤ 14 days	95% CI	*p* value
Univariable	Timing group only	182	0.61	0.42–0.90	0.0131
Model A	Age + sex	182	0.69	0.46–1.02	0.0613
Model B	Age + sex + KPS	142	0.61	0.38–0.98	0.0415
Model C	Model B + prescription dose + treated volume + fractionation	142	0.61	0.38–0.98	0.0430
Model D	Model C + extracranial disease status	142	0.62	0.38–1.00	0.0491
Model E	Model D + primary histology	142	0.58	0.35–0.97	0.0382

Cox proportional hazards models were used. KPS = Karnofsky Performance Status. Model D represents the primary fully adjusted model

In the primary fully adjusted model including age, sex, KPS at GKRS, prescription dose, treated volume, fractionation, and extracranial disease status, GKRS within 14 days remained associated with improved OS (HR, 0.62; 95% CI, 0.38–1.00; *p* = 0.0491). KPS at GKRS was independently associated with OS (HR, 0.97 per point increase; *p* = 0.0026), whereas extracranial disease status was not independently associated with OS (HR, 1.19; 95% CI, 0.72–1.97; *p* = 0.489). Adjustment for extracranial disease produced minimal attenuation of the association between early GKRS and OS. In an expanded model additionally adjusting for primary tumor histology, the association remained significant (HR, 0.58; 95% CI, 0.35–0.97; *p* = 0.0382). The association between GKRS within 14 days and improved OS persisted after additional adjustment for calendar year of surgery (adjusted HR 0.60, 95% CI 0.37–0.97; *p* = 0.037). Schoenfeld residual-based diagnostics did not identify significant departures from proportional hazards for the postoperative GKRS timing variable or the overall fully adjusted Cox model. A possible departure was observed for KPS at GKRS, although the global test was not significant.

### Sensitivity analysis excluding prolonged delays

Sensitivity analyses were performed to evaluate whether the association between early GKRS and OS was influenced by extreme treatment delays or by the choice of survival time origin.

First, patients with surgery-to-GKRS intervals greater than 60 days were excluded. After excluding these 10 patients, 172 patients remained. The OS difference associated with GKRS within 14 days persisted by log-rank testing (*p* = 0.0122). In univariable Cox regression, GKRS within 14 days remained associated with reduced mortality risk (HR, 0.61; 95% CI, 0.41–0.90; *p* = 0.0130). After adjustment for age, sex, KPS, prescription dose, treated volume, fractionation, and extracranial disease status, the effect estimate remained similar, although the confidence interval crossed unity (adjusted HR, 0.64; 95% CI, 0.40–1.04; *p* = 0.0711).

Second, OS was recalculated from the date of surgical resection rather than from the date of GKRS. Median OS was 38.2 months for patients treated within 14 days compared with 17.2 months for those treated after 14 days. The difference remained significant by log-rank testing (*p* = 0.0174). In univariable Cox regression, GKRS within 14 days was associated with reduced mortality risk (HR, 0.63; 95% CI, 0.43–0.92; *p* = 0.0183). After multivariable adjustment, the effect estimate remained similar but did not reach statistical significance (adjusted HR, 0.64; 95% CI, 0.39–1.02; *p* = 0.0624).

### Local recurrence-free survival

Local recurrence-free survival was evaluated in 111 patients with documented local recurrence or available post-GKRS intracranial imaging follow-up (Supplementary Figure 2). Of the 182 patients in the overall cohort, 71 lacked sufficient documented recurrence or imaging follow-up information for LRFS evaluation. Among the 111 evaluable patients, 50 experienced documented local recurrence and 61 were censored at their last available brain MRI. Using the 14-day threshold, LRFS did not differ significantly between patients treated within 14 days and those treated after 14 days by log-rank testing (*p* = 0.433). In univariable Cox regression, GKRS within 14 days was not significantly associated with LRFS (HR, 1.25; 95% CI, 0.71–2.19; *p* = 0.436). This finding remained non-significant after adjustment for age, sex, KPS at GKRS, prescription dose, treated volume, fractionation, and extracranial disease status (adjusted HR, 1.28; 95% CI, 0.66–2.49; *p* = 0.463). When patients were analyzed using three timing intervals of ≤ 14 days, 15–30 days, and > 30 days, LRFS distributions did not differ significantly across groups (*p* = 0.671).

Radiographically documented radiation necrosis was identified in 13 patients (7.1%) following GKRS. Ten patients (5.5%) had Grade 1 radiation necrosis and were managed conservatively with observation, whereas three patients (1.6%) required escalation of corticosteroid therapy. The precise CTCAE grade of the latter cases could not be reliably established retrospectively from the available clinical documentation.

## Discussion

In our cohort of 182 patients undergoing postoperative GKRS after brain metastasis resection, earlier radiosurgery was associated with improved OS. Outcome-oriented cutpoint analysis identified 14 days as the interval demonstrating the greatest observed OS separation, with directionally consistent findings across grouped timing comparisons, multivariable models, and sensitivity analyses excluding prolonged treatment delays or recalculating survival from surgery. However, the association did not retain statistical significance after adjustment for the cutpoint search, indicating that the precise 14-day threshold remains exploratory rather than definitively established. Patients treated within 14 days had substantially longer median OS than those treated later, whereas GKRS timing was not significantly associated with LRFS. Together, these findings suggest that postoperative GKRS timing may be a clinically meaningful component of the brain metastasis treatment pathway.

The optimal timing of postoperative SRS after brain metastasis resection remains unsettled [12, 13]. Some studies support early treatment, [8, 14] while others suggest that a short delay may allow postoperative recovery, cavity stabilization, improved target definition, or reduction in treatment-related toxicity [7,15–17]. Iorio-Morin et al. reported that delaying Gamma Knife treatment beyond three weeks was associated with increased local recurrence, supporting early postoperative radiosurgery [8]. Similar evidence linking longer postoperative delays with inferior local control has subsequently been reported.^14^ In contrast, Yaghi et al. suggested that initiating postoperative radiotherapy after 22 days was associated with reduced mortality, although delays beyond 40 days increased progression risk [9]. More recently, Bander et al. proposed a 22–30-day postoperative SRS window when balancing local failure and post-treatment radiation effect [10]. In our cohort, however, the longest median OS was observed with treatment within 14 days, and the 22–30-day interval did not demonstrate an apparent survival advantage. Although the four-group comparison did not reach statistical significance, the three-group analysis and cutpoint analysis both supported an early postoperative treatment window. These differences across studies may reflect variation in treatment platform, institutional workflow, primary endpoint selection, dose and fractionation strategy, patient selection, and definitions of postoperative radiation timing [12, 18].

The finding that early GKRS was associated with improved OS but not significantly improved LRFS is clinically important. LRFS was evaluable in a smaller imaging-follow-up cohort than OS, limiting power to detect modest local control differences. In addition, cavity-specific recurrence is only one of several factors influencing survival in metastatic disease. Contemporary prognostic models demonstrate that survival after brain metastasis diagnosis varies substantially according to primary tumor biology, performance status, extracranial disease, number of brain metastases, and molecular characteristics [19].

An important alternative explanation for the observed OS difference is selection bias related to postoperative recovery and fitness for early treatment. Patients who undergo GKRS within 14 days may represent a subgroup with more favorable postoperative trajectories, including fewer surgical complications, more rapid neurological recovery, and earlier readiness for oncologic treatment. Conversely, postoperative complications, wound-healing concerns, prolonged rehabilitation, or clinical deterioration may delay GKRS while independently contributing to poorer survival. Although preoperative KPS and performance status at GKRS did not differ significantly between timing groups, these measurements may not adequately capture differences in perioperative clinical fitness, postoperative complications, or the trajectory of neurological recovery. Consequently, the observed survival association may partly reflect differences in postoperative fitness rather than a direct effect of GKRS timing itself.

OS may therefore be affected by extracranial progression, distant intracranial failure, neurologic recovery, systemic therapy access, steroid dependence, functional decline, and ability to tolerate salvage therapy. In the present cohort, adjustment for extracranial disease status produced minimal attenuation of the association between early GKRS and OS, suggesting that the observed timing association was not explained solely by the presence of systemic metastatic disease at the time of GKRS. The absence of a statistically significant LRFS difference should not be interpreted as proof that timing has no biologic effect on the postoperative cavity. The discrepancy between OS and LRFS may reflect the influence of systemic disease, postoperative recovery, treatment selection, and other prognostic factors not fully captured by cavity-specific recurrence outcomes. This interpretation is consistent with our prior institutional work on longitudinal postoperative cavity remodeling, which showed that GKRS-treated cavities generally involute over time, but that volumetric change alone was not associated with local recurrence [20].

Several plausible mechanisms may underlie this association. Earlier GKRS may shorten the interval during which microscopic residual disease remains untreated in the surgical bed, potentially limiting subclinical progression before radiographic recurrence becomes measurable [8,14,15]. Earlier GKRS may facilitate completion of intracranial local therapy and potentially allow earlier initiation or resumption of systemic treatment, particularly in patients with extracranial metastatic disease or systemic therapy-sensitive histologies. However, systemic therapy type and timing were not systematically available in this cohort, and this proposed mechanism could not be directly evaluated. Differences in systemic treatment access or sequencing may also independently influence both GKRS timing and overall survival. Recent work examining sequencing of surgery, focal radiation, and extracranial therapy has demonstrated that shortening the postoperative radiation pathway can meaningfully reduce time to subsequent oncologic treatment [21]. This is clinically relevant because our prior data in renal cell and breast cancer brain metastases found systemic therapy to be associated with OS and to modulate intracranial disease [22, 23]. These potential mechanisms are not mutually exclusive and cannot be distinguished from recovery-related treatment selection in the present retrospective cohort. Earlier GKRS may therefore represent both a component of more efficient oncologic care and a marker of more favorable postoperative recovery, rather than an independently established determinant of improved survival.

The extended study period introduces additional potential confounding related to evolving systemic oncologic treatment. During the earlier years of the cohort, systemic treatment options were more limited, and delays in postoperative radiosurgery may have coincided with delays in initiating or resuming systemic therapy. In contemporary practice, targeted therapies and immunotherapy may be initiated during postoperative recovery, potentially altering the relationship between GKRS timing, systemic treatment sequencing, and survival. Although the association between earlier GKRS and OS persisted after adjustment for calendar year of surgery, calendar year cannot fully capture changes in systemic therapy exposure, tumor-specific treatment advances, or individual treatment sequencing. Accordingly, residual treatment-era confounding remains possible.

These findings have practical implications for postoperative care coordination. Although a causal survival benefit from earlier radiosurgery cannot be established from these retrospective data, the observed association supports attention to potentially avoidable delays in the postoperative treatment pathway. Early neurosurgical and radiation oncology coordination may facilitate timely postoperative imaging, cavity assessment, and GKRS planning while allowing treatment timing to be individualized according to neurological recovery, wound healing, and systemic oncologic priorities. Such coordination should not be interpreted as requiring GKRS within 14 days for every patient, but rather as supporting efficient multidisciplinary decision-making and timely treatment when clinically appropriate. Contemporary brain metastasis guidelines similarly emphasize integration of neurosurgery, radiation oncology, and systemic therapy when sequencing local treatment [13, 24].

Several strengths support the relevance of this study. The cohort represents a relatively large GKRS-specific postoperative brain metastasis series, allowing focused evaluation of Gamma Knife timing rather than pooling heterogeneous postoperative radiation approaches. The systematic evaluation of postoperative timing thresholds, supported by permutation adjustment, bootstrap resampling, and continuous spline modeling, enabled a comprehensive assessment of the observed association with early GKRS. Complementary grouped timing analyses demonstrated a consistent direction of association, while bootstrap resampling most frequently identified 14 days and showed that selected thresholds were concentrated within the early postoperative period. The association between early GKRS and improved OS was also evaluated across sequential multivariable models incorporating clinical and dosimetric covariates, including extracranial disease status, and showed similar effect estimates in sensitivity analyses excluding prolonged-delay outliers and recalculating OS from the date of surgery. Inclusion of treatment volume, dose, fractionation, performance status, histology, and extracranial disease allowed adjustment for several clinically relevant sources of prognostic heterogeneity.

This study has limitations. Its retrospective, single-institution design limits causal inference, and residual treatment-selection and guarantee-time biases cannot be excluded, particularly because the cohort included only patients who ultimately received GKRS. Although multivariable adjustment and sensitivity analyses accounted for several measured prognostic factors, postoperative complications, length of stay, steroid dependence, systemic therapy timing, and reasons for treatment delays were not systematically available. Consequently, differences in postoperative recovery, treatment readiness, and evolving systemic oncologic care may have contributed to the observed survival association. Additionally, some patients with prolonged treatment intervals may have experienced progression before their initial cavity-directed GKRS.

LRFS analyses were limited by incomplete imaging follow-up and competing mortality, precluding reliable competing-risk modeling and reducing statistical precision. Differentiation of local recurrence from radiation-related changes was based on available clinical and radiographic assessments, with advanced imaging techniques not uniformly available. Radiation necrosis was documented descriptively, but toxicity surveillance was not standardized. Finally, the 14-day threshold was derived from an outcome-oriented cutpoint search and did not retain statistical significance after permutation adjustment. Although bootstrap findings suggested an early postoperative timing signal, the precise optimal interval remains uncertain and requires external validation.

## Conclusion

Postoperative GKRS within 14 days of brain metastasis resection was associated with substantially longer overall survival, despite no significant difference in local recurrence-free survival. These findings position early GKRS as a potentially important component of postoperative oncologic care and highlight the need to minimize avoidable treatment delays. Timely multidisciplinary coordination and radiosurgical planning should therefore remain central priorities in postoperative brain metastasis care.

## Supplementary Information

Below is the link to the electronic supplementary material.

Supplementary Material 1

## Data Availability

The datasets generated during and/or analysed during the current study are available from the corresponding author on reasonable request.

## Abbreviations

CI: Confidence interval
ECOG: Eastern Cooperative Oncology Group
GKRS: Gamma Knife radiosurgery
HR: Hazard ratio
IQR: Interquartile range
KPS: Karnofsky Performance Status
LRFS: Local recurrence-free survival
MRI: Magnetic resonance imaging
NSCLC: Non-small cell lung cancer
OS: Overall survival
SRS: Stereotactic radiosurgery.
