# Supplementary material for: Does timing matter? Early postoperative Gamma Knife radiosurgery within 14 days is associated with longer overall survival after brain metastasis resection
Source: J Neurooncol. 2026 Nov 11;180(2):48. doi: 10.1007/s11060-026-05866-z (PMC13755093; doi:10.1007/s11060-026-05866-z)
Supplement: Supplementary file 1 [file 11060_2026_5866_MOESM1_ESM.docx]

**Supplementary Table 1. Local Recurrence-Free Survival Analysis.** LRFS = local recurrence-free survival. Timing was not significantly associated with LRFS.

| **Analysis** | **Result** |
| --- | --- |
| Derived LRFS cohort | 111 patients |
| Local recurrence events | 50 |
| Censored observations | 61 |
| LRFS log-rank p value, ≤14 vs >14 days | 0.433 |
| Univariable Cox HR for ≤14 days | 1.25 |
| 95% CI | 0.71–2.19 |
| Cox p value | 0.436 |
| Adjusted HR for ≤14 days | 1.28 |
| 95% CI | 0.66–2.49 |
| Adjusted p value | 0.463 |
| Three-group log-rank p value | 0.671 |

Supplementary Figures –

**Supplementary Figure 1. Overall survival by four postoperative GKRS timing intervals.** Kaplan–Meier curve comparing GKRS delivered ≤14 days, 15–21 days, 22–30 days, and >30 days after surgery. The global comparison across timing groups did not reach statistical significance (p=0.0939), although the longest median OS was observed among patients treated ≤14 days after surgery.

**Supplementary Figure 2. Local recurrence-free survival by postoperative GKRS timing.** Kaplan–Meier curve comparing patients treated ≤14 days versus >14 days after surgery. Timing was not significantly associated with local recurrence-free survival (log-rank p=0.433).

**Supplementary Figure 3. Overall survival from date of surgery by postoperative GKRS timing.** Kaplan–Meier curve comparing patients treated ≤14 days versus >14 days after surgery, with survival time calculated from surgical resection. Overall survival remained significantly different between timing groups when surgery was used as the time origin (log-rank p=0.0174).

**Supplementary Figure 4. Association between postoperative GKRS timing and overall survival using restricted cubic spline analysis.** Adjusted hazard ratios for all-cause mortality were estimated using Cox proportional hazards regression with a four-knot restricted cubic spline. The model adjusted for age, sex, Karnofsky Performance Status at GKRS, prescription dose, treated volume, fractionation, and extracranial disease status. The solid line represents the estimated hazard ratio relative to a surgery-to-GKRS interval of 14 days, and the shaded region represents the 95% confidence interval. The horizontal dashed line indicates a hazard ratio of 1.0, and the vertical dotted line marks the 14-day reference interval.
